# Assessment of brain structure and volume reveals neurodevelopmental abnormalities in preterm infants with low-grade intraventricular hemorrhage

**DOI:** 10.1038/s41598-024-56148-5

**Published:** 2024-03-08

**Authors:** Chunxiang Zhang, Zitao Zhu, Kaiyu Wang, Brianna F. Moon, Bohao Zhang, Yanyong Shen, Zihe Wang, Xin Zhao, Xiaoan Zhang

**Affiliations:** 1 Department of Radiology, The Third Affiliated Hospital of Zhengzhou University, Zhengzhou, China; 2 Institute of Neuroscience, Zhengzhou University, Zhengzhou, China; 3 Wuhan University, Wuhan, China; 4 GE Healthcare, MR Research China, Beijing, China; 5 Department of Radiology, Athinoula A. Martinos Center for Biomedical Imaging, Massachusetts General Hospital and Harvard Medical School, Boston, MA USA; 6 Zhengzhou University, Zhengzhou, China

**Keywords:** Low-grade intraventricular hemorrhage, Preterm Infants, Diffusion kurtosis imaging, Synthetic MRI, Neurodevelopment, Paediatric research, Predictive markers, Neuronal development

## Abstract

There is increasing evidence of abnormal neurodevelopmental outcomes in preterm infants with low-grade intraventricular hemorrhage (IVH). The purpose of the study was to explore whether brain microstructure and volume are associated with neuro-behavioral outcomes at 40 weeks corrected gestational age in preterm infants with low-grade IVH. MR imaging at term-equivalent age (TEA) was performed in 25 preterm infants with mild IVH (Papile grading I/II) and 40 control subjects without IVH. These subjects all had neonatal behavioral neurological assessment (NBNA) at 40 weeks’ corrected age. Microstructure and volume evaluation of the brain were performed by using diffusion kurtosis imaging (DKI) and Synthetic MRI. Correlations among microstructure parameters, volume, and developmental outcomes were explored by using Spearman's correlation. In preterm infants with low-grade IVH, the volume of brain parenchymal fraction (BPF) was reduced. In addition, mean kurtosis (MK), fractional anisotropy (FA), radial kurtosis (RK), axial kurtosis (AK) in several major brain regions were reduced, while mean diffusivity (MD) was increased (*P* < 0.05). BPF, RK in the cerebellum, MK in the genu of the corpus callosum, and MK in the thalamus of preterm infants with low-grade IVH were associated with lower NBNA scores (r = 0.831, 0.836, 0.728, 0.772, *P* < 0.05). DKI and Synthetic MRI can quantitatively evaluate the microstructure alterations and brain volumes in preterm infants with low-grade IVH, which provides clinicians with a more comprehensive and accurate neurobehavioral assessment of preterm infants with low-grade IVH.

## Introduction

Although the survival rate of preterm infants has improved over the years, brain injury in preterm infants gives rise to adverse neurodevelopmental disorders^1^. Intraventricular hemorrhage (IVH) is a characteristic form of neonate brain injury^2^. IVH is classified through the Papile grading system with the level of severity ranging across four grades^3^. Although the incidence of IVH has decreased, the prevalence continues to rise with improved detection methods^4,5^. In particular, severe IVH is associated with increased short-term neurological morbidity^6^, while the short-term neurological outcome of low—grade IVH is still debated and remains an active area of research^7,8^.

Research has shown that the microstructure of corpus callosum, limbic pathways and cerebellum can be changed in premature infants with low-grade IVH^9^. At present, diffusion tensor imaging (DTI) is often used in detecting brain microstructure of IVH, including observing white matter (WM) microstructural changes in preterm neonates with mild germinal matrix-intraventricular hemorrhage (GMH-IVH). In addition, DTI can be used to assess the relationship between microstructural differences and early motor function, and cerebellar maturation^4,8,9^. However, traditional DTI, based on the hypothesis of gaussian motion of water molecules, deviates from the actual water motion. It cannot solve the problem of cross-fiber orientation in brain tissue, and is not robust in exploring anisotropic structure such as gray matter^10^. Development of diffusion kurtosis imaging (DKI) has introduced an advanced quantitative imaging technology to mitigate these limitations. DKI provides kurtosis parameters including mean kurtosis (MK), axial kurtosis (AK) and radial kurtosis (RK). Both diffusion and kurtosis parameters are considered to reflect the heterogeneity of the diffusion environment within the tissue. In recent years, DKI has been used to evaluate brain development^11^. MK allows a more sensitive evaluation of age-related microstructural changes in both WM and gray matter (GM) and is potentially a valuable technique for studying brain development^12^. DKI has shown a potential advantage in detecting the normal brain development of infants^13^.

IVH can also cause a decrease in brain volume, and it is related to the adverse effects of the neuropsychological outcome in preterm infants^14,15^. Synthetic MRI is an emerging technology with fast acquisition for multiple quantitative maps and user-friendly brain volume segmentation and calculation by using a commercial post-processing software. Meanwhile, Synthetic MRI has been confirmed the accessibility of robust synthetic volumetric results through a clinical workstation^16^ and a study^17^ has substantiated the alignment of Synthetic MRI brain tissue volumetry with the established brain growth curve.

Neonatal behavioral neurological assessment (NBNA) is designed to test neonatal behavior and allow early detection of mild brain damage for early intervention. It refers to five areas: behavioral capacity, passive muscle tone, active muscle tone, primitive reflex, and general assessment, with a total of 20 items on a 40-point scale. Moreover, recent studies have found that NBNA provides diagnostic assessment of brain damage in premature infants and animal models^18,19^.

DKI combined with Synthetic MRI has not yet been used to explore microstructural changes in the brain of preterm infants with low-grade IVH. Therefore, the aim of the current study was to explore whether brain microstructure and volume at term-equivalent age (TEA) measured by DKI and synthetic MRI are associated with NBNA scores at the same developmental stage in low-grade IVH infants.

## Methods

### Participants

**Figure 1:**
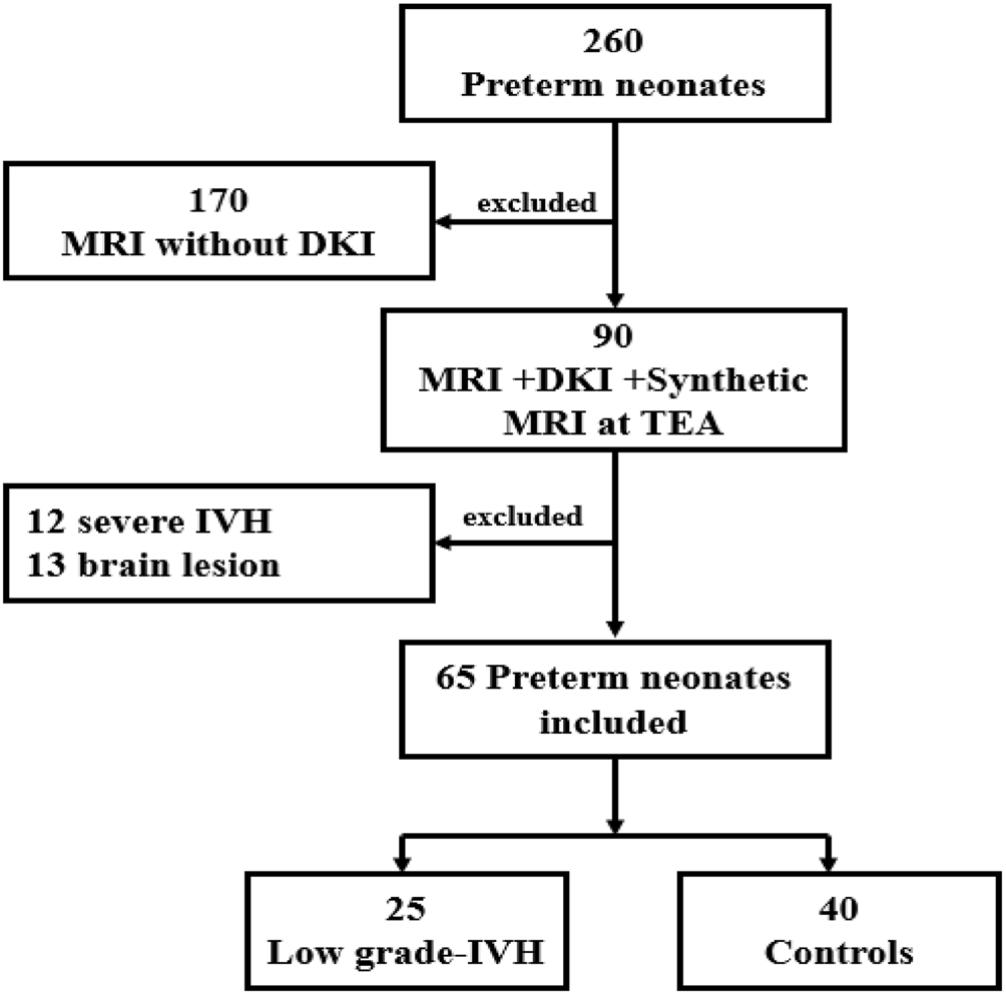
Flow-chart showing the study group. TEA, term- equivalent age; DKI, diffusion kurtosis imaging; IVH, intraventricular hemorrhage.

In this retrospective study, medical records were reviewed for information regarding low-grade IVH diagnosis and other pertinent perinatal/clinical variables. Premature neonates underwent an early brain ultrasound scan at the first post-natal week and a routine single-session MRI examination including DKI and Synthetic MRI sequences. Then at the 40 weeks these premature babies received NBNA test. The severity of GMH-IVH has been graded according to the Papile classification system:

Grade I—subependymal hemorrhage, Grade II—IVH without ventricular dilatation, Grade III—IVH with ventricular dilatation, and Grade IV—IVH with parenchymal hemorrhage^3^. According to this standard, we include preterm infants with low-grade IVH (grade I-II). Three exclusion criteria were then considered: (i) presence of severe IVH (grade III- IV), (ii) presence of brain lesions other than low-grade IVH, and (iii) MRI images with motion artifacts. In total there were 25 cases in the low-grade IVH group and 40 cases in the healthy group. Details are shown in Fig. 1.

Ethics Committee of the Third Affiliated Hospital of Zhengzhou University approved this retrospective study and the parents of all the study participant provided written informed consent. All experiments were performed in accordance with the Declaration of Helsinki.

### MRI acquisition and processing

**Figure 2:**
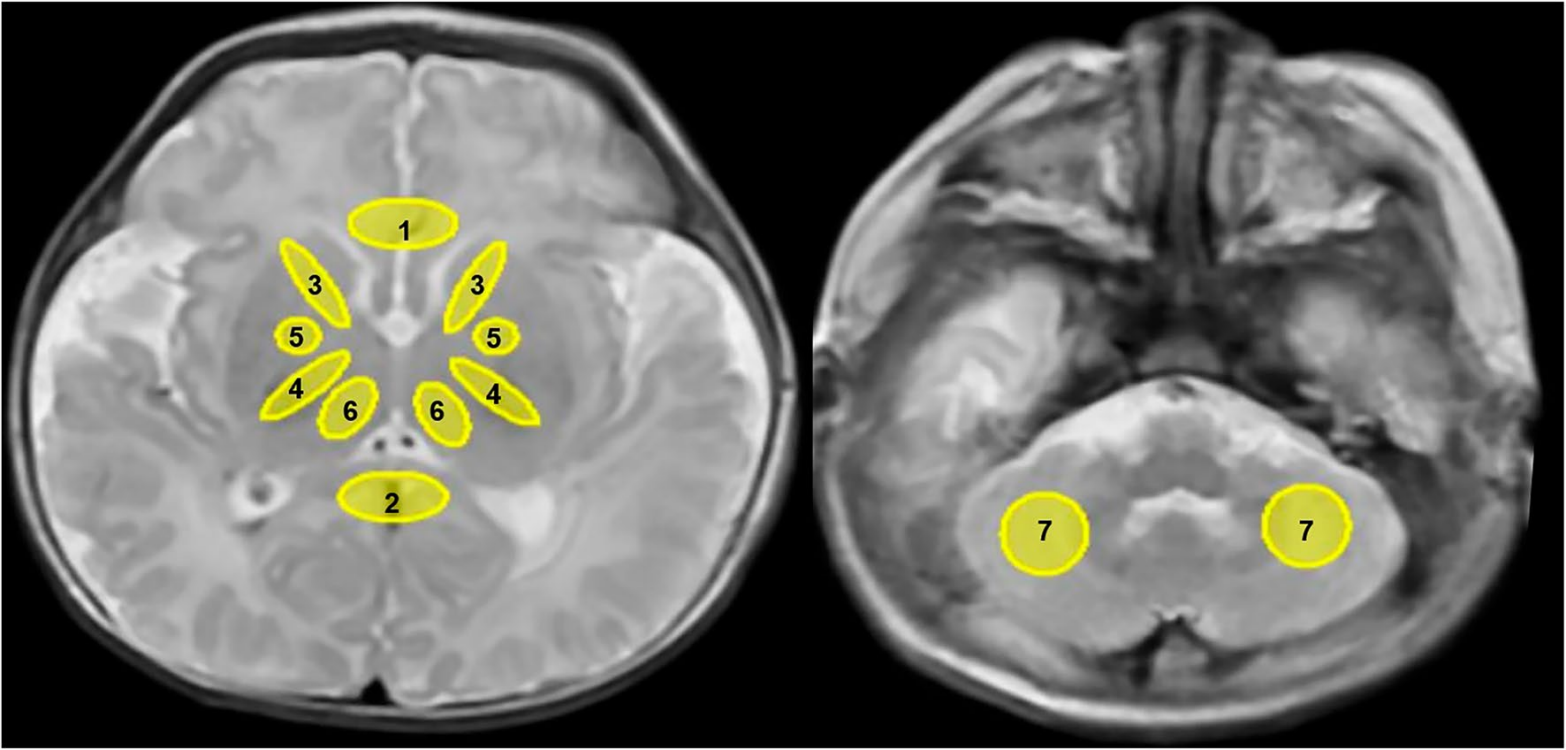
Positions of regions of interest (ROIs). Elliptical ROIs are drawn on the T_2_WI map. 1 genu of the corpus callosum (GCC), 2 splenium of the corpus callosum (SCC), 3 anterior limbs of internal capsule (ALIC), 4 posterior limbs of the internal capsule (PLIC), 5 globus pallidum (GP), 6 thalamus (TH) and 7 cerebellum.

All studies were performed on a 3.0 T MR imaging scanner (Pioneer, GE Healthcare, Milwaukee, WI) with an eight-channel, phased-array head coil. Routine scan sequences T_1_-weighted imaging (T_1_WI) and T_2_-weighted imaging (T_2_WI) were obtained. DKI sequence parameters were TR = 2000 ms, TE = 2.32 ms, the number of diffusion directions is set to 30 for b-values of 1000 and 2000 mm^2^/s, respectively. In addition, slice thickness = 3.0 mm without a gap, acquisition matrix = 96 × 96, reconstruction matrix = 256 × 256 and acquisition time = 7.5 min. Despite the DKI acquisition matrix size being 96 × 96, the reconstruction pipeline automatically interpolates to an image size of 256 × 256. Then MK, RK, AK, fractional anisotropy (FA), mean diffusivity (MD) maps were generated from DKI images on vendor-supplied post-processing workstation. Meanwhile, we use ITK-SNAP 4.0 (http://www.itksnap.org/pmwiki/pmwiki.php) to draw ROI. ITK-SNAP provides semi-automatic segmentation using active contour methods, as well as manual delineation and image navigation. In addition to these core functions, ITK-SNAP also offers many other supporting utilities. Seven regions of interest (ROI) were outlined on slices of basal ganglia and cerebellum, including posterior limbs of the internal capsule (PLIC), anterior limbs of internal capsule (ALIC), genu of the corpus callosum (GCC), splenium of the corpus callosum (SCC), thalamus (TH), globus pallidum (GP), and cerebellum (Cere). The ROI size was controlled within 10 ± 5 mm^2^. The ROIs were delineated manually by two radiologists (with 8 and 7 years of experience in reading images and diagnosis, respectively). Two radiologists who were blind to the clinical data did the measurement separately. Details are shown in Fig. 2.

Dedicated synthetic MR imaging (SyMRI) software (Synthetic MR, Linköping, Sweden) (Version 11.2; https://www.syntheticmr.com/) facilitates the reconstruction of synthetic images with a combination of virtually any TE, TR, and TI. SyMRI also provides fully automated volumetric analysis based on the expected quantitative values for different brain tissue types, and brain volume analysis is available in less than 1 min. Quantitative maps serve as input for automatic segmentation, which has been reported to be precise and robust^16,17^. In addition, SyMRI algorithmically eliminates issues related to coil sensitivity, RF non-uniformity and contrast differences across the imaging volume. Additional information regarding the software can be found in the published article by Akifumi Hagiwara et al.^20^. Parameters for synthetic MRI were as follows: slice thickness = 3.0 mm, field of view = 220 mm × 186 mm, scan time = 4 min. The neonatal brain exhibits higher hydration levels compared to an adult brain, leading earlier SyMRI versions to detect a cerebrospinal fluid (CSF) haze throughout the entire brain. In this study, we utilized a prototype of SyMRI (Version 11.2; https://www.syntheticmr.com/) specifically tailored for fast and precise volumetry in neonates. The Version 11.2 effectively suppresses the CSF contribution in the immature brain, converting the previously detected erroneous CSF into gray matter (GM) as the white matter (WM) definition remains at adult values. The volume of cerebrospinal fluid (CSF), and brain parenchymal fraction were determined as the metrics. Additional details are available in S1.

### Assessment of developmental outcome

The neurodevelopmental assessment was performed at 40 weeks of corrected gestational age by using neonatal behavioral neurological assessment (NBNA)^4^. The NBNA scoring system included five major items, behavioral ability, passive muscle tone, active muscle tone, primary reflexes, and general assessment. In detail it included 20 sub-items with 2 points assigned to each item and a total of 40 points. An NBNA score below 37 is considered abnormal.

### Statistical analysis

Statistical tests were performed on the Statistical Package for Social Sciences (IBM Corp., Armonk, NY, USA) software for Windows version 20.0. The demographics, DKI parameters, brain volumes, and NBNA scores between the low-grade IVH group and the control group were compared by using the chi-square test for qualitative variables, student’s t-test for normal distribution quantitative variables, and the Mann–Whitney U-test for non-normal distribution quantitative variables. False discovery rate (FDR) for multiple comparisons. MR images including DKI and Synthetic MRI of 25 preterm infants with mild IVH (Papile grading I/II) and 40 control subjects without IVH were used to assess inter-observer consistency by two radiologists. The parameters showed good inter-observer agreement as displayed in Table 1. And the model of ICC is Two-Way Mixed Effects. Spearman correlation analysis was performed to examine the relationship between DKI parameters, brain volumes, and NBNA test results. *P* values < 0.05 indicated statistical significance.

**Table 1:** Inter-observer consistency of measurements.

Parameters	Intraclass correlation coefficient, 95% CI
FA	0.955 (0.866–0.985)
MD	0.860 (0.630–0.963)
MK	0.943 (0.756–0.982)
RK	0.914 (0.756–0.973)
AK	0.914 (0.744–0.971)

95% CI = 95% confidence interval.

Fractional anisotropy, FA; mean diffusivity, MD; mean kurtosis, MK; radial kurtosis, RK; axial kurtosis, AK.

## Results

### Participants

We recruited 240 preterm infants, including 65 preterm infants who underwent DKI, Synthetic MRI acquisitions, and NBNA tests. Exclusions included brain lesions other than IVH, severe IVH, or not attending or completing the developmental assessments.

Significant differences were not detected between infants with low-grade IVH and the control subjects in GA, birth weight, postmenstrual age, and body weight at MR imaging as shown in Table 2.

**Table 2:** Baseline characteristics of the cohort.

Characteristic	Low-grade IVH (n = 25)	No IVH (n = 40)	*P* value
male/female ^a^	11/14	12/18	0.765
natural birth /C-section^a^	3/12	9/13	0.842
GA at birth (weeks)^b^	30.92 ± 2.16	30.85 ± 2.01	0.881
BW (g)^b^	1397.32 ± 238.36	1484.03 ± 348.95	0.239
Apgar 1 min^b^	6 ± 1.68	6.1 ± 1.65	0.814
Apgar 5 min^b^	6.64 ± 1.55	7 ± 1.16	0.833
PMA at MRI (weeks)^b^	39.01 ± 1.01	39.07 ± 1.19	0.901
BW at MRI (g)^b^	2652.40 ± 277.51	2677.90 ± 257.90	0.708
Days of intubation (days)	31 (14–38)	27 (5–29)	0.051
Hydrocortisone treatment (n)	7	11	0.52
Single pregnancy/multiple pregnancy^a^	20/5	31/9	0.811
Maternal educational background: High school diploma /No^a^	8/17	13/27	0.967
Tobacco consumption during pregnancy/No^a^	11/14	19/21	0.783

GA, gestational age; PMA, post-menstrual age; BW, birth weight.

(a) with X^2^ test; (b) with t-test.

### Quantitative measurements of brain locations and volumes

**Figure 3:**
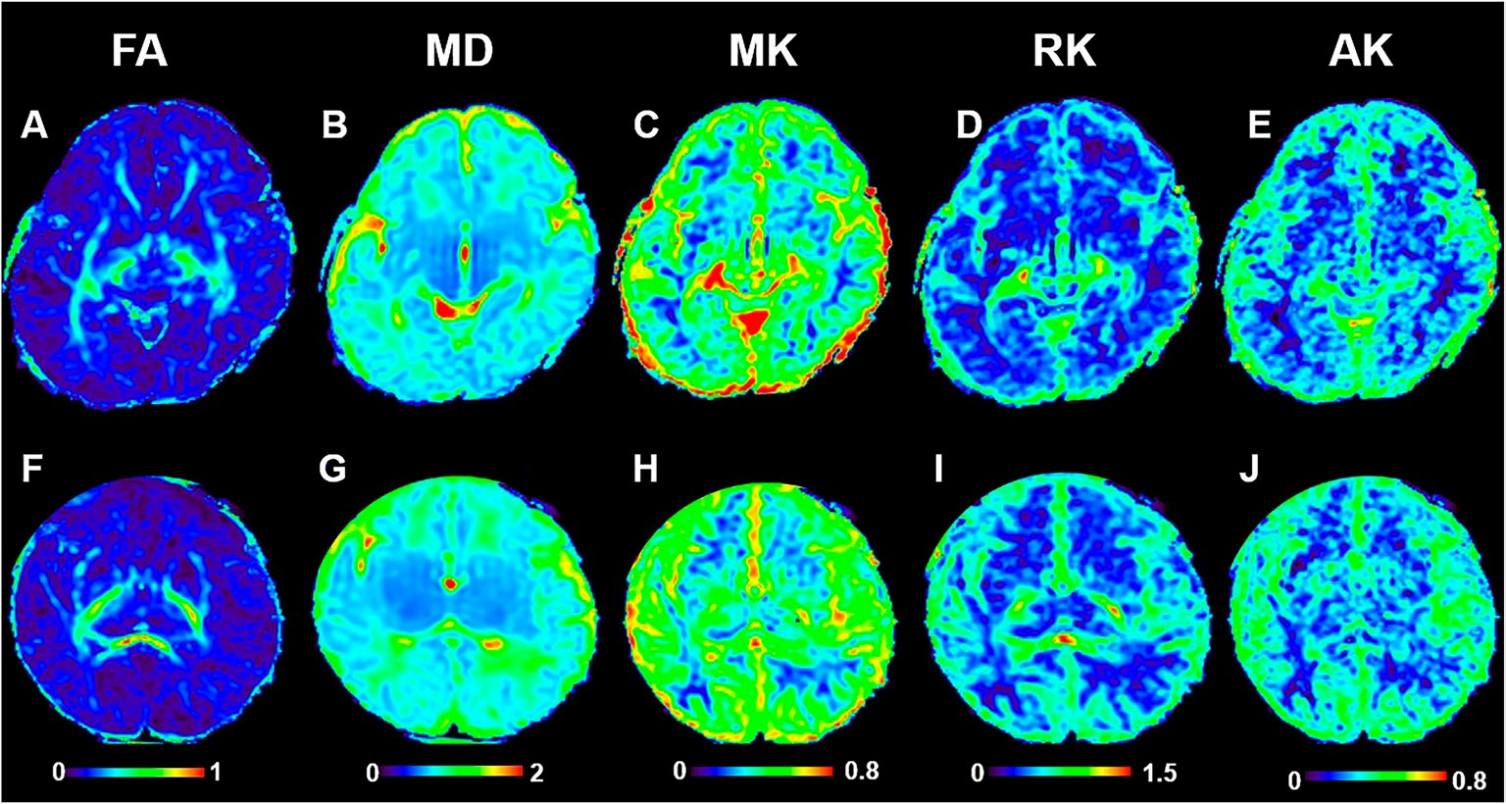
DKI images of preterm infant brain with low-grade IVH and without IVH. FA, MD, MK, RK and AK images of preterm infant brain without low-grade IVH (**A**–**E**) and with low-grade IVH (**F**–**G**). The two cases were all male, 40 weeks. fractional anisotropy, FA; mean diffusivity, MD; mean kurtosis, MK; radial kurtosis, RK; axial kurtosis, AK.

Figure 3 displayed FA, MD, MK, RK and AK images of preterm infant brain with low-grade IVH(A–E) and without IVH (F–G). The two cases were selected from the disease group (preterm infant brain with low-grade IVH) and the healthy group. They are all male, 38 weeks.

**Figure 4:**
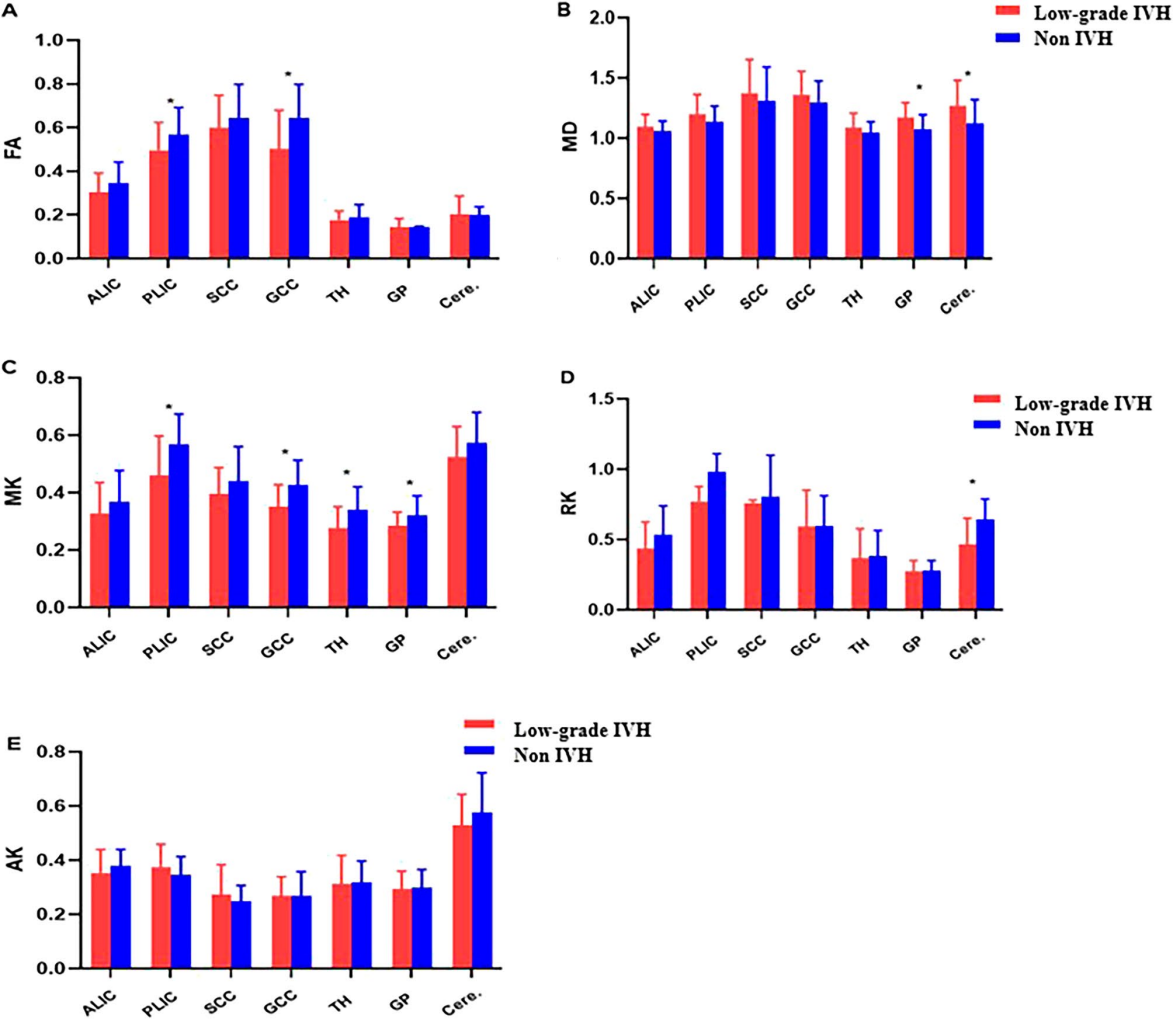
Comparing the differences of parameters between low-grade IVH (n = 25) and control group (n = 40) in different brain regions. Red represented preterm infants with low-grade IVH, blue represented Non—IVH group. (**A**) FA values between low-grade IVH and non-IVH in all ROIs. (**B**) MD values between low-grade IVH and non-IVH in all ROIs. (**C**) MK values between low-grade IVH and non-IVH in all ROIs. (**D**) RK values between low-grade IVH and non-IVH in all ROIs. (**E**) AK values between low-grade IVH and non-IVH in all ROIs. *P < 0.05. *Q value < 0.1. Fractional anisotropy, FA; mean diffusivity, MD; mean kurtosis, MK; radial kurtosis, RK; axial kurtosis, AK. Anterior limbs of internal capsule, ALIC; posterior limbs of the internal capsule, PLIC; splenium of the corpus callosum, SCC; genu of the corpus callosum, GCC; thalamus, TH; globus pallidum, GP; cerebellum, Cere.

As shown in Fig. 4, significant differences were found between low-grade preterm infants and the control group. In the low-grade IVH group, the FA value (0.496 ± 0.127 vs. 0.568 ± 0.122, *P* = 0.025) and MK value (0.460 ± 0.136 vs. 0.570 ± 0.194, *P* = 0.016) of PLIC were significantly different from those in the control group. The FA and MK values of GCC in the control group were significantly higher than those in the low-grade preterm infant group (0.606 ± 0.119 vs. 0.500 ± 0.180, *P* = 0.012; 0.426 ± 0.086 vs. 0.351 ± 0.075, *P* < 0.001). MK in TH and GP were all significantly different from that of the control group (0.275 ± 0.074 vs. 0.339 ± 0.079, *P* = 0.020; 0.285 ± 0.046 vs. 0.321 ± 0.067,* P* = 0.024, respectively). There is a significant difference in the RK of the cerebellum between the two groups (0.464 ± 0.185 vs. 0.643 ± 0.144, *P* < 0.001). The MD values of the GP was significantly higher than that in the control group (1.170 ± 0.125 vs. 1.075 ± 0.120, *P* = 0.030). The MD values of the cerebellum showed a trend of higher in the patient group than in the control group (1.267 ± 0.212 vs. 1.121 ± 0.196, *P* = 0.040).

**Figure 5:**
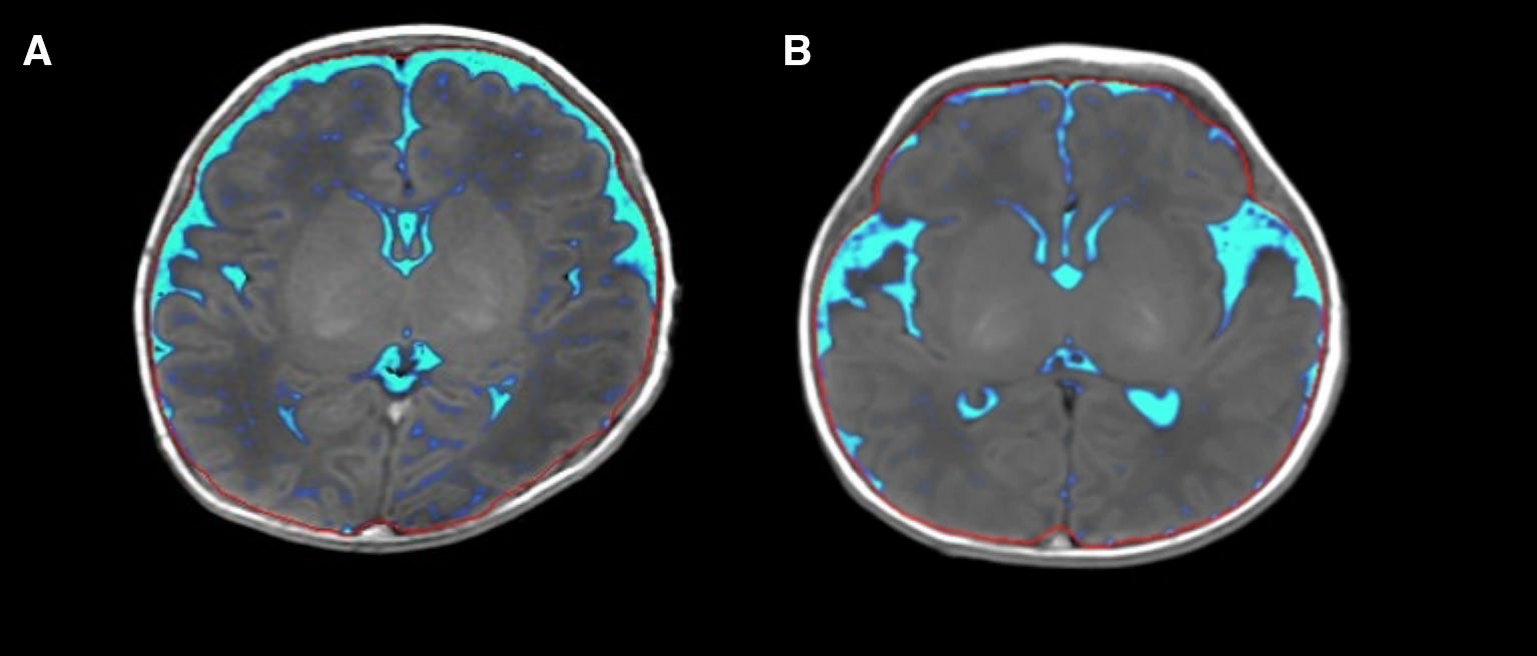
The volume of CSF in preterm infant brain with low-grade IVH (**A**) and without low-grade IVH (**B**). The two cases were all male, 38 weeks. Brain volume segmentation by Synthetic MRI. Cerebrospinal fluid, CSF.

The volume results of brain tissue segmentation in the two groups were shown in Table 3 and Fig. 5. The CSF volume in the low-grade IVH group was higher than that in the control group but without significant difference (84.660 ± 21.355 vs. 75.580 ± 21.270, P = 0.200). Moreover, the BPF in the low-grade IVH group was significantly lower than that in the control group (0.760 ± 0.065 vs. 0.760 ± 0.065, *P* < 0.001).

**Table 3:** Comparison of brain volume between groups.

	Low-grade IVH (n = 25)	Non IVH (n = 40)	*P* value	Q value
CSF	84.660 ± 21.355 ml	75.580 ± 21.270 ml	0.200	0.200
BPF	0.760 ± 0.065	0.861 ± 0.033	< 0.001*	0.002*

Cerebrospinal fluid, CSF.

Brain Parenchymal Fraction (BPF) = Brain Parenchymal Volume/Intracranial volume.

*P < 0.05. *Q value < 0.1.

### NBNA outcome

**Figure 6:**
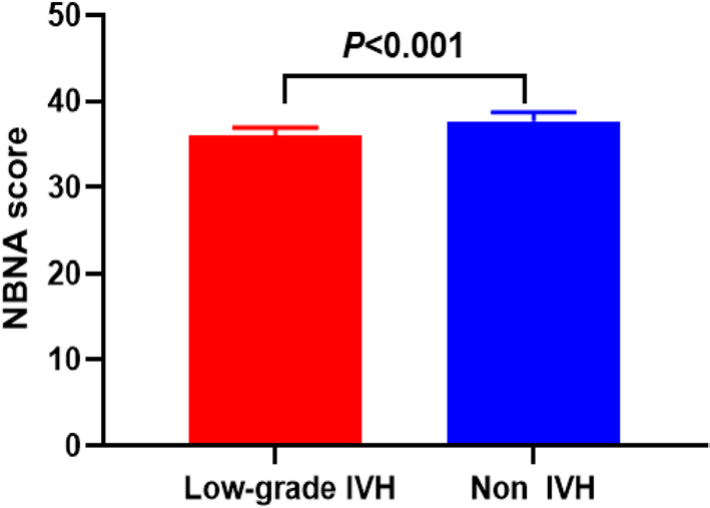
Neurodevelopmental outcome of the low-grade IVH group and the non-IVH group.

The NBNA score in the low-grade premature infant group were significantly lower than those in the control group (*P* < 0.05), as shown in Fig. 6

### Correlations of DKI parameters and volumes with NBNA outcome

**Figure 7:**
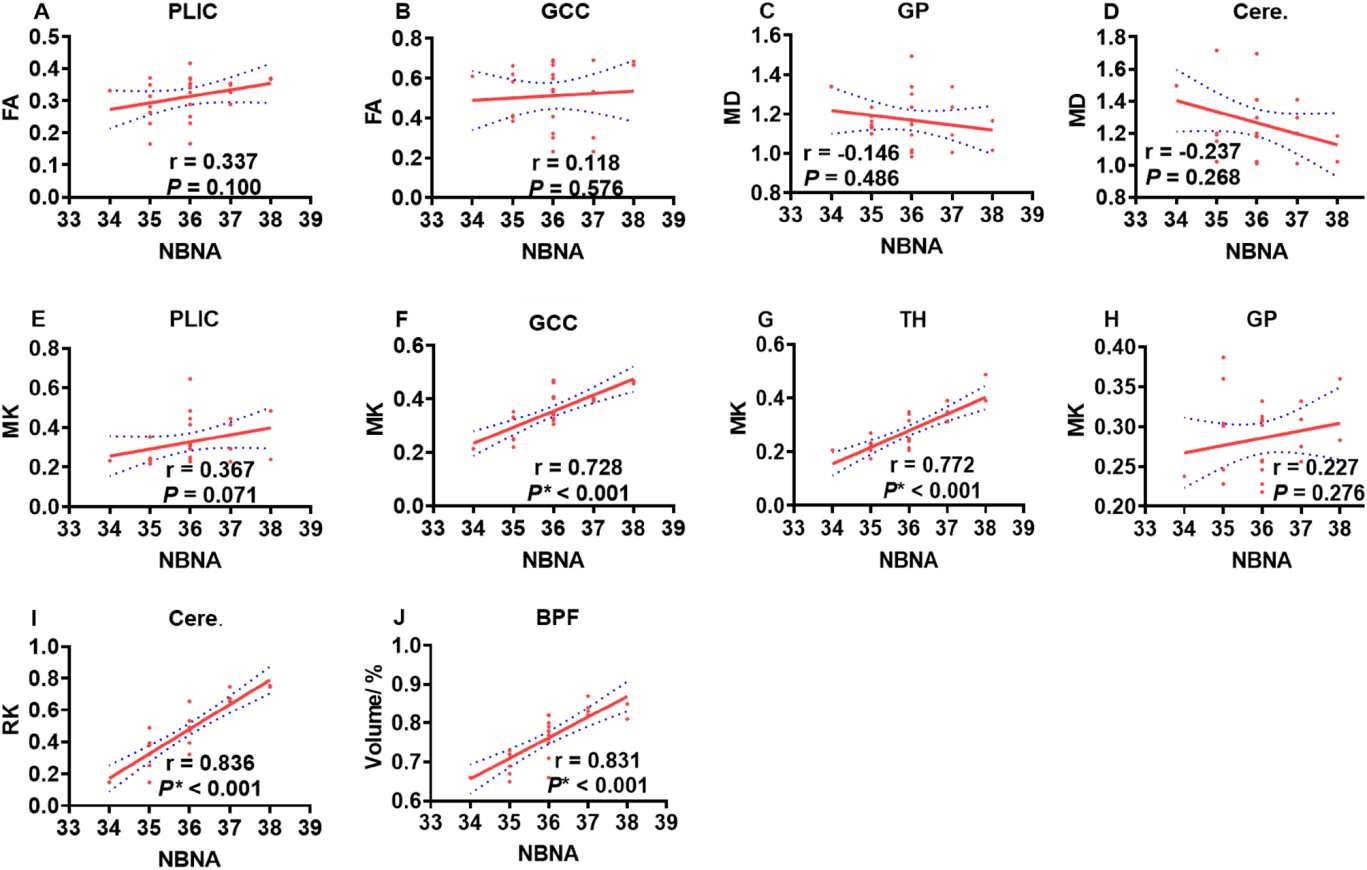
Diffusion parameters related to NBNA scores. The graph (**A**, **B**) demonstrates the relationship between FA in PLIC/GCC and NBNA. The graph (**C**, **D**) demonstrates the relationship between MD in GP/Cere. and NBNA. The graph (**E**–**H**) demonstrates the relationship between MK in PLIC/GCC/TH/GP and NBNA. The graph(I) demonstrates the relationship between RK in Cere. and NBNA. The graph(J) demonstrates the relationship between BPF and NBNA. Fractional anisotropy, FA; mean diffusivity, MD; mean kurtosis, MK; radial kurtosis, RK. Posterior limbs of the internal capsule, PLIC; genu of the corpus callosum, GCC; thalamus, TH; globus pallidum, GP; cerebellum, Cere; brain parenchymal fraction, BPF.

Based on the above results, we performed Spearman correlation analysis. MK in the GCC was positively correlated with NBNA (r = 0.728, *P* < 0.01). Similarly, MK value in the TH and RK in the cerebellum were positively correlated with NBNA (r = 0.772 and 0.836, respectively, *P* < 0.001); BPF were all positively correlated with NBNA (r = 0.831, respectively, *P* < 0.001), as shown in Fig. 7.

## Discussion

IVH is the most common pattern of brain injury in preterm infants^21,22^. We found that MK in the GCC and the TH, RK in the Cerebellum, and BPF are significantly correlated with neurological outcome at 40 weeks of low-grade IVH preterm.

Our findings indicate decreased MK and FA in the PLIC and the GCC and increased MD in the GP and cerebellar in preterm infants with low-grade IVH. This is in line with previous studies^8,23–25^, in which smaller brain areas showed decreased FA value and increased MD in the IVH preterm. Premature infants also showed lower MK values than full-term infants^13,26,27^. Preterm infants with low-grade IVH showed relatively low FA and MK in the GCC which may be attributed to the reduced bulk density of fiber tracts after brain injury. Interestingly, we found that the diffusion kurtosis parameter MK can show the difference of deep nuclei (TH, globus pallidus) between the two groups, while the diffusion parameter FA does not show the difference in anisotropic tissue.

In addition, there were different volume changes of BPF between low-grade IVH and the control. Previous studies have shown that brain volume is reduced in preterm infants with IVH^28^. Premature infants have reduced gray matter and total brain volume compared to healthy full-term infants^29^, and relatively increased cerebrospinal fluid volume^30^. Our results demonstrated that BPF were reduced in premature infants with low-grade IVH.

Some evidence suggests that the IVH may cause a low NBNA score^18^. Neonates with IVH are at greater risk of impaired neurodevelopmental outcomes than those without IVH. This is consistent with our previous study in which lower scores are present in preterm infants with low-grade IVH^31^. IVH is associated with increased short-term and long-term neurological morbidity^32^. The advantage of NBNA is that it can detect minor brain damage at an early stage, which is conducive to early intervention.

The present study demonstrates that there are strong correlations between NBNA score and DKI parameters including MK in the GCC, MK in the TH, and RK in the cerebellum. This is consistent with previous research^18,33^. In IVH preterm infants, the microstructure of several regions including the corpus callosum, internal capsule and superior longitudinal fasciculus at TEA are related to cognitive and/or behavior outcomes at age 40 weeks^34^. The thalamus is the high-level sensory center, and it will be at risk for sensory problems and dyskinesias when damaged^35,36^. In addition, our research indicates a correlation between RK in cerebellum and NBNA, as demonstrated in previous studies^4,37^.

This study has some limitations, primarily the small sample size. Larger cohorts are required to extend and validate this study. Secondly, some studies suggest that IVH is often accompanied by changes in cerebellar volume. However, we can only measure the whole brain segmentation volume here and detect the abnormal development characteristics of microstructure in the cerebellum. In addition, the small ROI size might lead to slice selection bias in our study. Finally, this batch of research subjects is age-limited, and we are currently unable to detect neurodevelopmental scores at the age of 1–2 years. We plan to assess the score at 1 year, when differences between the low-grade IVH and non-IVH children would be more apparent than at 40 weeks.

## Conclusion

DKI and Synthetic MRI can quantitatively evaluate the brain injury of premature infants with low-grade IVH. Smaller brain BPF and altered microstructure at TEA were associated with poorer brain development. Meanwhile, MK values show a greater change than others after GM injury, suggesting that MK is sensitive in evaluating GM damage.

### Supplementary Information

Supplementary Information.

## Data Availability

The datasets used and/or analyzed during the current study available from the corresponding author on reasonable request. A submission to the journal implies that materials described in the manuscript, including all relevant raw data, will be freely available to any researcher wishing to use them for non-commercial purposes, without breaching participant confidentiality.
