# Supplementary material for: Assessment of brain structure and volume reveals neurodevelopmental abnormalities in preterm infants with low-grade intraventricular hemorrhage
Source: Sci Rep. 2024 Mar 8;14:5709. doi: 10.1038/s41598-024-56148-5 (PMC10923809; doi:10.1038/s41598-024-56148-5)
Supplement: Supplementary file 1 — Supplementary Information. [file 41598_2024_56148_MOESM1_ESM.docx]

**S1 MRI Acquisition Protocol for preterm neonate**

| **Parameter** | **T1WI** | **T2WI** | **DKI** | **Synthetic MRI** |
| --- | --- | --- | --- | --- |
| Field of view (mm) | 200 x 200 | 200 x 200 | 256 x 256 | 220 x186 |
| Matrix | 320 x 320 | 320 x 320 | 96 x 96 | 288 x 224 |
| Repetition time (msec) | 3010 | 4500 | 2000 | 4000 |
| Echo time (msec) | 14 | 99 | 2.32 | 19.6 |
| Inversion time (mse) | - | - | - | 28.1 |
| Number of averages | 1 | 1 | 1 | 1 |
| Flip angle (degrees) | 12 | 12 | 90 | 90 |
| Section thickness/gap | 4/0 | 4/0 | 3/0 | 3/0 |
| Number of directions | NA | NA | 30 | NA |
| Acquisition time | 2 min | 2 min | 7.5 min | 4 min |
